# Modelling of plant circadian clock for characterizing hypocotyl growth under different light quality conditions

**DOI:** 10.1093/insilicoplants/diac001

**Published:** 2022-02-02

**Authors:** Miao Lin Pay, Dae Wook Kim, David E Somers, Jae Kyoung Kim, Mathias Foo

**Affiliations:** 1 Institute for Future Transport and Cities, Coventry University, Coventry CV1 2TE, UK; 2 Department of Mathematical Sciences, Korea Advanced Institute of Science and Technology, Daejeon 34141, Republic of Korea; 3 Biomedical Mathematics Group, Institute for Basic Science, Daejeon 34126, Republic of Korea; 4 Department of Molecular Genetics, The Ohio State University, Columbus, OH 43210, USA; 5 Center for Applied Plant Sciences, The Ohio State University, Columbus, OH 43210, USA; 6 School of Engineering, University of Warwick, Coventry CV4 7AL, UK

**Keywords:** *Arabidopsis thaliana*, competitive binding, hypocotyl growth, light qualities, photoperiodic growth, plant circadian system

## Abstract

To meet the ever-increasing global food demand, the food production rate needs to be increased significantly in the near future. Speed breeding is considered as a promising agricultural technology solution to achieve the zero-hunger vision as specified in the United Nations Sustainable Development Goal 2. In speed breeding, the photoperiod of the artificial light has been manipulated to enhance crop productivity. In particular, regulating the photoperiod of different light qualities rather than solely white light can further improve speed breading. However, identifying the optimal light quality and the associated photoperiod simultaneously remains a challenging open problem due to complex interactions between multiple photoreceptors and proteins controlling plant growth. To tackle this, we develop a first comprehensive model describing the profound effect of multiple light qualities with different photoperiods on plant growth (i.e. hypocotyl growth). The model predicts that hypocotyls elongated more under red light compared to both red and blue light. Drawing similar findings from previous related studies, we propose that this might result from the competitive binding of red and blue light receptors, primarily Phytochrome B (phyB) and Cryptochrome 1 (cry1) for the core photomorphogenic regulator, CONSTITUTIVE PHOTOMORPHOGENIC 1 (COP1). This prediction is validated through an experimental study on *Arabidopsis thaliana*. Our work proposes a potential molecular mechanism underlying plant growth under different light qualities and ultimately suggests an optimal breeding protocol that takes into account light quality.

## 1. INTRODUCTION

The rapid population growth across the world has increased global food demand. According to the United Nations Food and Agricultural Organization (FAO), food production will have to increase 70 % by 2050 to feed the global population, and keeping up with this increasing demand remains a challenge for the global agricultural sector (Alexandratos and Bruinsma 2012). In particular, crops yield and quality have been threatened by adverse weather conditions such as long periods of drought, hurricane, flood together with ever decreasing available cultivable land area (see, e.g. Ahmad *et al.* 2009; Mar *et al.* 2018; Pareek *et al.* 2020). In order to increase crop production sustainably, innovative technological solutions such as speed breeding are becoming necessary (Ghosh *et al.* 2018; Li *et al.* 2018; Watson *et al.* 2018; Chiurugwi *et al.* 2019).

Speed breeding is a state-of-the-art agricultural technology in smart agricultural, which accelerates the plant development by manipulating the environmental condition. The pioneering work by Watson *et al.* (2018) developed a protocol in manipulating the photoperiod of artificial light, which can increase about 2-fold of crop productivity compared to conventional breeding method. While the speed breeding method has mainly been conducted under white LED lights, utilizing different light qualities rather than the white light can potentially further promote plant growth and development (see, e.g. Kami *et al.* 2010; Manivannan *et al.* 2017; Ajdanian *et al.* 2019). As such, we ask ourselves the following questions: can we achieve even better crop productivity using speed breeding protocol by optimizing the photoperiod of these different light qualities? To answer the above questions involving inherent complexity of the interplay between photoreceptors and downstream proteins regulating plant growth, we need a combination of experimental and *in silico* approaches. Specifically, we need a mathematical model that describes the effect of multiple light qualities on plant growth and development. Given this purpose, the model of plant circadian system (PCS) is promising as the PCS is responsible for the manipulation of multiple physiological processes including resource efficient plant growth and development (Hotta 2021; Lochocki and McGrath 2021).

Indeed, PCS models including the mechanism of plant growth and development have been recently proposed (Seaton *et al.* 2015; De Caluwé *et al.* 2016). In particular, the model proposed by De Caluwé *et al.* (2016) is of interest. The authors grouped and merged several clock genes into single component according to the similar nature of their behaviours resulting in a compact PCS model. Furthermore, to ensure this compact model is able to capture PCS-controlled growth, they included the hypocotyl growth. The hypocotyl is the seedling stem located above the root and underneath the seed leaves. As the hypocotyl is the main growing part of plant towards light stimulus, it can act as a relevant proxy for our investigation relating light qualities to plant growth.

Despite the aforementioned progress in PCS modelling, almost all the PCS models available considered regulation involving only white light. To the best of our knowledge, the only study that incorporated light quality into PCS model was carried out in Ohara *et al.* (2015a), where the authors replaced the commonly used light-responsive protein P proposed in Locke *et al.* (2005) with three photoreceptors, namely Phytochrome A (phyA), Phytochrome B (phyB) and Cryptochrome 1 (cry1), which are sensitive to red and blue light, respectively. As a mean of model validation, the authors showed that the model was able to reproduce experimental Phase Response Curve (PRC) under different light quality. Due to the different scope of study, their model did not consider any direct interactions between the photoreceptors and a core photomorphogenetic regulator, the CONSTITUTIVE PHOTOMORPHOGENIC1/SUPPRESSOR OF PHYA-105 E3 ligase complex (COP1/SPA; termed here COP1) nor any direct relationship between light quality and plant growth.

In this study, we developed a mathematical model for PCS demonstrating the effect of various light quality conditions on plant growth. Specifically, we incorporated the light quality function from Ohara *et al.* (2015a) into the compact model proposed in De Caluwé *et al.* (2016) to develop a PCS model for characterizing hypocotyl growth under different light quality conditions. To account for plant growth through photomorphogenesis and skotomorphogenesis and their interactions with the photoreceptors (Kim *et al.* 2017; Lymperopoulos *et al.* 2018), we included, for the first time, the interaction of a light signalling centre, COP1, with photoreceptors (Lau and Deng 2012; Su *et al.* 2017) into our model. Using this model, we predicted that the possibility of competitive binding among the photoreceptors for COP1 (Liu *et al.* 2011; Podolec and Ulm 2018; Lau *et al.* 2019; Paik *et al.* 2019) could lead to more hypocotyl elongation under red light than under both red and blue light, or blue alone. This prediction was confirmed with our follow-up experiments of hypocotyl measurements under different light quality conditions across several photoperiods. The model can act as a first contribution towards an extensive mathematical model describing other plant organs growth under different light quality conditions. This complements the resource- and time-consuming experimental analysis with *in silico* simulations for determining not only the optimal photoperiod for different light qualities, but potentially other light properties that can be exploited to enhance crop productivity.

## 2. MODEL DESCRIPTIONS

### 2.1 Model development

We have adopted a compact model of PCS introduced by De Caluwé *et al.* (2016) that has been widely used for various computational analysis due to its simplicity and accuracy (see, e.g. Tokuda *et al.* 2019; Greenwood *et al.* 2020). The model consists of 12 ordinary different equations (ODEs), where the core circadian system is represented by eight ODEs, the effect of light with a light-sensitive protein is represented by one ODE and the genetic component that regulates hypocotyl growth is represented by three ODEs.

The core circadian genes are labelled as: CL (CCA1 and LHY), P97 (PSEUDO RESPONSE REGULATOR 9 and 7 (PRR9 and PRR7)), EL (EARLY FLOWERING 4 (ELF4) and LUX ARRHYTHMO (LUX)) and P51 (PRR5 and TOC1). Following Locke *et al.* (2005), the light-sensitive protein is represented by protein P. The genetic component associated with the hypocotyl growth is labelled PHYTOCHROME INTERACTING FACTOR 4 and 5 (PIF4 and PIF5) and the hypocotyl length is denoted by HYP.

We have made several modifications to the model. First, the activation of P97 by CL protein is modified to repression based on recent studies (Fogelmark and Troein 2014; Adams *et al.* 2015), which showed that CL protein represses the expression of all other clock genes including P97. Second, to investigate the effect of red and/or blue lights on the hypocotyl growth, the single light-sensitive protein is replaced by three photoreceptors; phyA, phyB and cry1 following the approach described in Ohara *et al.* (2015a) (see the following section for more detailed description of the light module). Thus, the revised PCS model is represented by the following ODEs:

*Core PCS:*


$$\begin{array}{l} \begin{array}{ll} \frac{d{\left[CL\right]}_{m}}{dt}= & (v_{1}+L_{a})\;\left(\frac{1}{1+{\left(\frac{{\left[P97\right]}_{p}}{K_{1}}\right)}^{2}+{\left(\frac{{\left[P51\right]}_{p}}{K_{2}}\right)}^{2}}\right)\\ & -(k_{1L}\Theta_{PhyA}+k_{1D}(1-\Theta_{PhyA})){\left[CL\right]}_{m} \end{array} \end{array}$$ (1)

$$\begin{array}{l} \frac{d{\left[CL\right]}_{p}}{dt}=(p_{1}+p_{1L}\Theta_{PhyA})\;{\left[CL\right]}_{m}-d_{1}\;[CL] \end{array}$$ (2)

$$\begin{array}{l} \begin{array}{ll} \frac{d{\left[P97\right]}_{m}}{dt}= & (v_{2}+L_{b})\;\left(\frac{1}{1+{\left(\frac{{\left[CL\right]}_{p}}{K_{3}}\right)}^{2}+{\left(\frac{{\left[P51\right]}_{p}}{K_{4}}\right)}^{2}+{\left(\frac{{\left[EL\right]}_{p}}{K_{5}}\right)}^{2}}\right)\\ & -k_{2}{\left[P97\right]}_{m} \end{array} \end{array}$$ (3)

$$\begin{array}{l} \frac{d{\left[P97\right]}_{p}}{dt}=p_{2}{\left[P97\right]}_{m}-(d_{2D}(1-\Theta_{PhyA})+d_{2L}\Theta_{PhyA}){\left[P97\right]}_{p} \end{array}$$ (4)

$$\begin{array}{l} \frac{d{\left[P51\right]}_{m}}{dt}=\frac{v_{3}}{1+{\left(\frac{{\left[CL\right]}_{p}}{K_{6}}\right)}^{2}+{\left(\frac{{\left[P51\right]}_{p}}{K_{7}}\right)}^{2}}-k_{3}{\left[P51\right]}_{m} \end{array}$$ (5)

$$\begin{array}{l} \frac{d{\left[P51\right]}_{p}}{dt}=p_{3}{\left[P51\right]}_{m}-(d_{3D}(1-\Theta_{PhyA})+d_{3L}\Theta_{PhyA}){\left[P51\right]}_{p} \end{array}$$ (6)

$$\begin{array}{l} \frac{d{\left[EL\right]}_{m}}{dt}=\frac{\Theta_{PhyA}v_{4}}{1+{\left(\frac{{\left[CL\right]}_{p}}{K_{8}}\right)}^{2}+{\left(\frac{{\left[P51\right]}_{p}}{K_{9}}\right)}^{2}+{\left(\frac{{\left[EL\right]}_{p}}{K_{10}}\right)}^{2}}-k_{4}{\left[EL\right]}_{m} \end{array}$$ (7)

$$\begin{array}{l} \frac{d\;{\left[EL\right]}_{p}}{dt}=p_{4}{\left[EL\right]}_{m}-(d_{4D}(1-\Theta_{PhyA})+d_{4L}\Theta_{PhyA}){\left[EL\right]}_{p} \end{array}$$ (8)

*Hypocotyl growth:*


$$\begin{array}{l} \frac{d{\left[PIF\right]}_{m}}{dt}=\frac{v_{5}}{1+{\left(\frac{{\left[EL\right]}_{p}}{K_{11}}\right)}^{2}}-k_{5}{\left[PIF\right]}_{m} \end{array}$$ (9)

$$\begin{array}{l} \frac{d{\left[PIF\right]}_{p}}{dt}=p_{5}{\left[PIF\right]}_{m}-(d_{5D}(1-\Theta_{PhyA})+d_{5L}\Theta_{PhyA}){\left[PIF\right]}_{p} \end{array}$$ (10)

$$\begin{array}{l} \frac{d{\left[HYP\right]}_{p}}{dt}=g_{1}+\frac{g_{2}[PIF{]}_{p}^{2}}{K_{12}^{2}+[PIF{]}_{p}^{2}} \end{array}$$ (11)

*Photoreceptor and light functions:*


$$\begin{array}{l} \frac{d[PhyA]}{dt}=(1-\Theta_{PhyA})A_{p3}-\frac{A_{m7}[PhyA]}{A_{k7}+[PhyA]}-q_{2}\Theta_{PhyA}[PhyA] \end{array}$$ (12)

$$\begin{array}{l} \frac{d[PhyB]}{dt}=B_{p4}-\frac{B_{m8}[PhyB]}{B_{k8}+[PhyB]} \end{array}$$ (13)

$$\begin{array}{l} \frac{d[Cry1]}{dt}=C_{p5}-\frac{C_{m9}[Cry1]}{C_{k9}+[Cry1]} \end{array}$$ (14)

$$\begin{array}{ll} L_{u}= & \;q_{1u}[PhyA]\Theta_{PhyA}+q_{3u}[PhyB]log(\eta_{1}I_{red}+1)\Theta_{PhyB}\\ & +q_{4u}[Cry1]log(\eta_{2}I_{blue}+1)\Theta_{Cry1} \end{array}$$ (15)

where ${\left[\;j\right]}_{k}$ is the dimensionless concentrations of the plant genes, and index k=m,p represents to the mRNA or protein. Index j denotes the genes CCA/LHY (CL), P97 (PRR9/PRR7), P51 (PRR5/TOC1), EL (ELF4/LUX), phyA, phyB, cry1 and PIF/PIF4 (PIF). $L_{u}$, u∈{aorb} is the effect of light input, $I_{red}$ and $I_{blue}$ are the red and blue light intensity, η is the normalization parameter of light intensity and the parameters $\Theta_{PhyA},\;\Theta_{PhyB}$ and $\Theta_{Cry1}$ are given by


$$\begin{array}{l} \Theta_{PhyA}=\left\{ \begin{array}{ll} 1, & I_{red}\;or\;I_{blue}\neq 0,\\ 0, & otherwise \end{array}\right. \end{array}$$

$$\begin{array}{l} \Theta_{PhyB}=\left\{ \begin{array}{ll} 1, & I_{red}\neq 0,\\ 0, & otherwise \end{array}\right. \end{array}$$

$$\begin{array}{l} \Theta_{Cry1}=\left\{ \begin{array}{ll} 1, & I_{blue}\neq 0,\\ 0, & otherwise \end{array}\right. \end{array}$$

### 2.2 Light quality-dependent competitive binding mechanism

**Figure 1.**
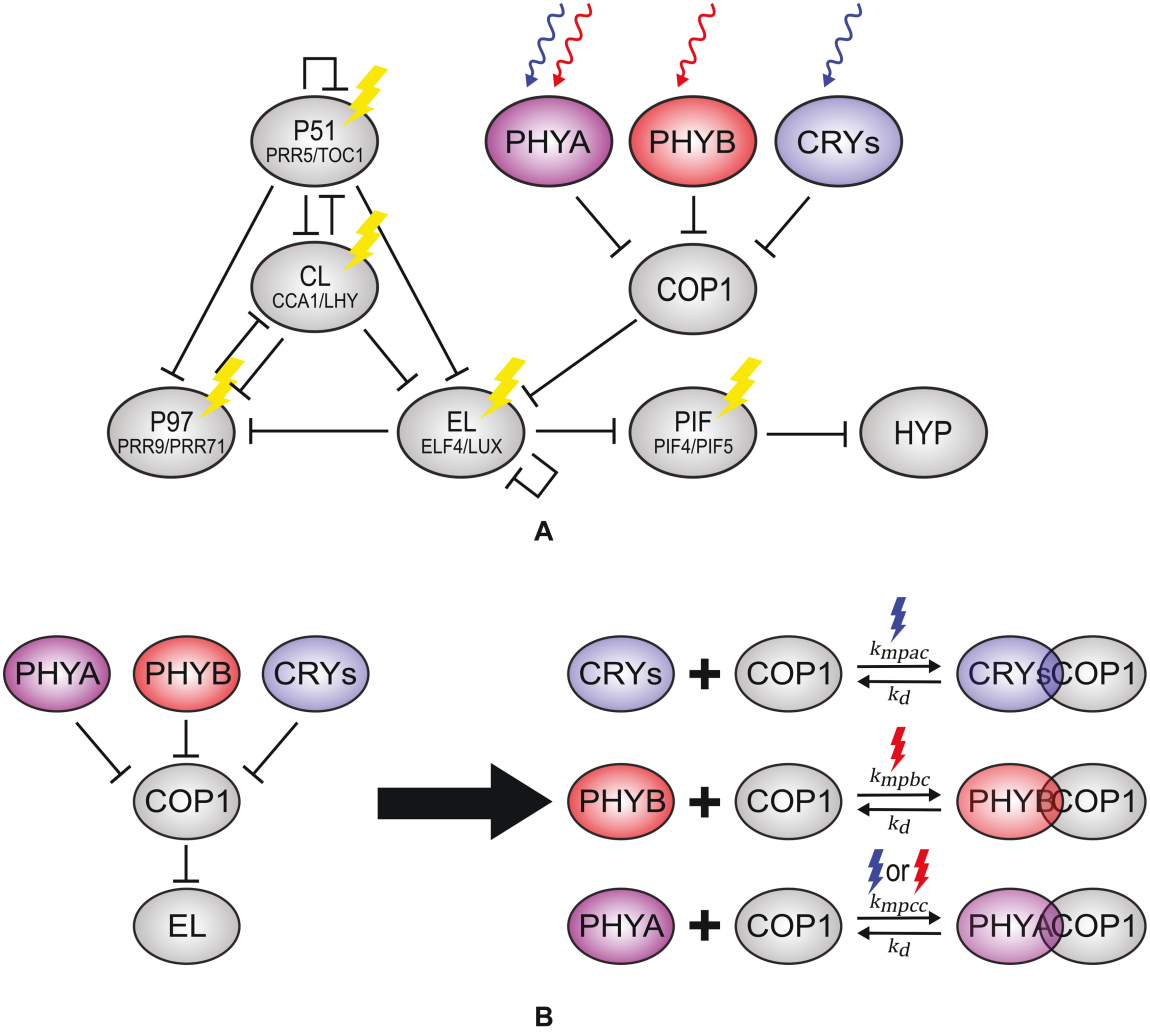
(A) Overview of the model developed in this work. (B) Competitive binding between COP1 and photoreceptors.

According to Lau and Deng (2012) and Han *et al.* (2020), COP1 is a key regulator of photomorphogenesis and acts as a central switch for the light-responsive proteins in *Arabidopsis thaliana*. Specifically, the physical interaction between COP1/SPA complex and cryptochrome and phytochrome photoreceptors inhibits COP1/SPA activity (Wang *et al.* 2001; Yang *et al.* 2001; Lian *et al.* 2011; Liu *et al.* 2011; Lu *et al.* 2015; Sheerin *et al.* 2015; Paik *et al.* 2019). We added this light quality-dependent interaction between COP1 and the photoreceptors to reflect that when plants are exposed to red and blue light simultaneously, phyA, phyB and cry1 are all activated, potentiating their concurrent binding with COP1. We hypothesize the possibility of a form of ‘competition’ between receptors for COP1/SPA occupancy when both red and blue lights are on simultaneously (Fig. 1A).

The competitive binding hypothesis considered in our work is supported largely by a previous finding that under blue light, activated cryptochrome will compete with COP1 substrates for COP1–WD binding through valine–proline (VP) motifs (Lau *et al.* 2019). This mechanism protects the downstream transcription factors from ubiquitination and thus promotes photomorphogenesis under blue light. Nevertheless, the interaction between COP1 and phytochromes was not reported in that study.

However, when they are activated by light, phytochromes and cryptochromes can disrupt the COP1/SPA interaction through direct binding to the C-terminal region of SPA, which includes a coiled-coil and WD domain (Liu *et al.* 2011; Paik *et al.* 2019). This inhibits the activity of COP1/SPA complex. In our experiments, co-illumination with blue and red light means both phytochromes and cryptochromes are activated. Since these photoreceptors bind to the same region of SPA1, competitions between the photoreceptors may plausibly occur. This would be likely even if the binding sites are not identical but adjacent, with the presence of one photoreceptor restricting the access of the other. As well, if activated phytochrome (i.e. Pfr) and activated cry1 differ significantly in their affinity for SPA1 association, it is conceivable that under certain light intensities and ratios of red and blue light the sum of their effects will not be additive, with greater shortening than in red alone, but longer hypocotyls than in blue alone. Instead, the more effective photoreceptor–SPA1 interaction (e.g. cry1–SPA1) might be diminished by the presence of the less effective interactor (e.g. phy–SPA1). This motivated us to take into account the possible effect of competitive binding in our model when both light qualities are present. This mechanism is illustrated in Fig. 1B.

Furthermore, COP1 is involved in the ubiquitination and degradation of both phyA and phyB (Seo *et al.* 2004; Jang *et al.* 2010), the rate of which may also be light intensity-dependent. These facts, combined with additional mechanisms of hypocotyl growth control via interactions with these photoreceptors and the Phytochrome Interacting Factors (PIFs) (Su *et al.* 2017) underlie the complexity of clearly delineating all the interactions the govern stem growth.

It is notable that competitive binding mechanisms have been reported in the other plant clock molecules (i.e. PRRs, TOC1, ZTL and GI) (Más *et al.* 2003; Kim *et al.* 2007; Para *et al.* 2007; McClung and Gutiérrez 2010). For example, PRR3 and ZTL competitively bind to TOC1 (Más *et al.* 2003; Para *et al.* 2007). This plays a critical role in regulating the stability of TOC1, resulting in the higher amplitude of rhythmicity of the protein. Furthermore, competitive binding mechanisms have also been reported in the mammalian circadian clock (Mitsui *et al.* 2001; Uriu and Tei 2017; Yoshitane *et al.* 2019).

To incorporate the competitive interactions between COP1 with the photoreceptors in our model, we modified the ODE for COP1 taken from Pokhilko *et al.* (2012) as follows:


$$\begin{array}{l} \begin{array}{ll} & \frac{d[COP1]}{dt}=-k_{mpac}\Theta_{PhyA}[PhyA][COP1]+k_{d}[COP1:PhyA]\\ & \;\;\;\;\;\;\;\;\;\;\;\;\;\;\;\;\;\;\;\;\;\;\;\;\;+k_{d}[COP1:PhyB]-k_{mpbc}\Theta_{PhyB}[PhyB][COP1]\\ & \;\;\;\;\;\;\;\;\;\;\;\;\;\;\;\;\;\;\;\;\;\;\;\;\;\;-k_{mpcc}\Theta_{Cry1}[Cry1][COP1]+k_{d}[COP1:Cry1]\\ & \;\;\;\;\;\;\;\;\;\;\;\;\;\;\;\;\;\;\;\;\;\;\;\;\;\;+\frac{A_{m7}[COP1:PhyA]}{A_{k7}+[COP1:PhyA]}+q_{2}\Theta_{PhyA}[COP1:PhyA]\\ & \;\;\;\;\;\;\;\;\;\;\;\;\;\;\;\;\;\;\;\;\;\;\;\;\;\;\;+\frac{B_{m8}[COP1:PhyB]}{B_{k8}+[COP1:PhyB]}+\frac{C_{m9}[COP1:Cry1]}{C_{k9}+[COP1:Cry1]} \end{array} \end{array}$$ (16)

$$\begin{array}{l} \begin{array}{ll} & \frac{d[COP1:PhyA]}{dt}=k_{mpac}\Theta_{PhyA}[PhyA][COP1]-k_{d}[COP1:PhyA]\\ & \;\;\;\;\;\;\;\;\;\;\;\;\;\;\;\;\;\;\;\;\;\;\;\;\;\;\;\;\;\;\;\;\;\;\;\;\;\;\;\;\;-\frac{A_{m7}[COP1:PhyA]}{A_{k7}+[COP1:PhyA]}-q_{2}\Theta_{PhyA}[COP1:PhyA] \end{array} \end{array}$$ (17)

$$\begin{array}{ll} \frac{d[COP1:PhyB]}{dt}=\; & k_{mpbc}\Theta_{PhyB}[PhyB][COP1]-k_{d}[COP1:PhyB]\\ & -\frac{B_{m8}[PhyB]}{B_{k8}+[PhyB]} \end{array}$$ (18)

$$\begin{array}{ll} \frac{d[COP1:Cry1]}{dt}=\; & k_{mpcc}\Theta_{Cry1}[Cry1][COP1]-k_{d}[COP1:Cry1]\\ & -\frac{C_{m9}[Cry1]}{C_{k9}+[Cry1]} \end{array}$$ (19)

where $k_{mpac}$, $k_{mpbc}$ and $k_{mpcc}$ are the binding rates of phyA, phyB and cry1, respectively, $k_{d}$ refers to the rate of dissociation and the notation : represents complex binding.

Since the photoreceptors are binding to COP1, we need to modify the photoreceptors Equations (13–15), as follows:


$$\begin{array}{l} \begin{array}{ll} & \frac{d[PhyA]}{dt}=(1-\Theta_{PhyA})A_{p3}-\frac{A_{m7}[PhyA]}{A_{k7}+[PhyA]}-q_{2}\Theta_{PhyA}[PhyA]\\ & \;\;\;\;\;\;\;\;\;\;\;\;\;\;\;\;\;\;\;\;\;\;\;\;\;-k_{mpac}\Theta_{PhyA}[PhyA][COP1]+k_{d}[COP1:PhyA] \end{array} \end{array}$$ (20)

$$\begin{array}{ll} \frac{d[PhyB]}{dt}=\; & B_{p4}-\frac{B_{m8}[PhyB]}{B_{k8}+[PhyB]}-k_{mpbc}\Theta_{PhyB}[PhyB][COP1]\\ & +k_{d}[COP1:PhyB] \end{array}$$ (21)

$$\begin{array}{ll} \frac{d[Cry1]}{dt}=\; & C_{p5}-\frac{C_{m9}[Cry1]}{C_{k9}+[Cry1]}-k_{mpcc}\Theta_{Cry1}[Cry1][COP1]\\ & +k_{d}[COP1:Cry1] \end{array}$$ (22)

Then, the total concentration of photoreceptors are given by


$$\begin{array}{l} \left[T_{PhyA}]=[PhyA]+[COP1:PhyA\right] \end{array}$$ (23)

$$\begin{array}{l} \left[T_{PhyB}]=[PhyB]+[COP1:PhyB\right] \end{array}$$ (24)

$$\begin{array}{l} \left[T_{Cry1}]=[Cry1]+[COP1:Cry1\right] \end{array}$$ (25)

Following the above modifications, the light input Equation (15) needs to be modified as well and this is given by:


$$\begin{array}{l} \begin{array}{ll} L_{u}= & q_{1u}([T_{PhyA}])\Theta_{PhyA}+q_{3u}([T_{PhyB}])log(\eta_{1}I_{red}+1)\Theta_{PhyB}\\ & +q_{4u}([T_{Cry1}])log(\eta_{2}I_{blue}+1)\Theta_{Cry1} \end{array} \end{array}$$ (26)

In addition, COP1 ubiquitin E3 ligase was also found to degrade the evening components, EL activity (Yu *et al.* 2008). As such, Equation (8) is modified as:


$$\begin{array}{ll} \frac{d{\left[EL\right]}_{p}}{dt}= & p_{4}{\left[EL\right]}_{m}-de_{1}{\left[EL\right]}_{p}\\ & -\left(\frac{de_{2}[COP1]+de_{3}[COP1:PhyA]}{\left[C_{tot}\right]}\right)\;{\left[EL\right]}_{p}\\ & -\left(\frac{de_{4}[COP1:PhyB]+de_{5}[COP1:Cry1]}{\left[C_{tot}\right]}\right)\;{\left[EL\right]}_{p} \end{array}$$

where $C_{tot}=[COP1]+[COP1:PhyA]+[COP1:PhyB]+[COP1:Cry1]$ is the total concentration of COP1.

### 2.3 Parameter estimation

The modified model consists of 18 ODEs with 66 parameters **[see****Supporting Information—Table S1****]**. The values of the two parameters, $\eta_{1}$ and $\eta_{2}$, which were used to scale the experimental light intensity were directly adopted from Ohara *et al.* (2015a). The value of the total COP1 concentration, $C_{tot}$, was set at 1 for simplicity and due to the lack of experimentally measured profiles of COP1. The values of the remaining parameters were estimated through optimization by minimizing a cost function, the weighted mean-squared error of the simulated proteins and mRNAs with their respective reference profiles (see Section 3 for details).

We used the reference profiles simulated by the two models proposed in De Caluwé *et al.* (2016) and Ohara *et al.* (2015a) since our model was developed through the incorporation of the two models. Specifically, the reference profiles of CL, P97, P51, EL and PIF were generated from De Caluwé *et al.* (2016), and the reference profiles of phyA, phyB and cry1 were generated from Ohara *et al.* (2015a).

## 3. MATERIALS AND METHODS

### 3.1 Model simulation and parameter estimation

All model simulations were performed using the ode15s solver in MATLAB. The model consists of 18 ODEs with 66 parameters. The initial concentration of all the mRNA and protein of the clock genes were set to one except the protein complexes and hypocotyl growth, which were set to zero. The light intensities normalization parameters $\eta_{1}$ and $\eta_{2}$ were adopted from Ohara *et al.* (2015a). The other 63 parameters were estimated through optimization using MATLAB function fminsearch by minimizing the cost function, e which is given below:


$$\begin{array}{l} \begin{array}{ll} & e=\sum_{i=1}^{N}\frac{{\left([CL{]}_{m,p}^{*}(i)-{\left[CL\right]}_{m,p}(i)\right)}^{2}}{N\times max\left([CL{]}_{m,p}^{*}\right)}+\sum_{i=1}^{N}\frac{{\left([P97{]}_{m,p}^{*}(i)-{\left[P97\right]}_{m,p}(i)\right)}^{2}}{N\times max\left([P97{]}_{m,p}^{*}\right)}\\ & \;\;\;\;\;+\sum_{i=1}^{N}\frac{{\left([P51{]}_{m,p}^{*}(i)-{\left[P51\right]}_{m,p}(i)\right)}^{2}}{N\times max\left([P51{]}_{m,p}^{*}\right)}+\sum_{i=1}^{N}\frac{{\left([EL{]}_{m,p}^{*}(i)-{\left[EL\right]}_{m,p}(i)\right)}^{2}}{N\times max\left([EL{]}_{m,p}^{*}\right)}\\ & \;\;\;\;\;+\sum_{i=1}^{N}\frac{{\left([PIF{]}_{m,p}^{*}(i)-{\left[PIF\right]}_{m,p}(i)\right)}^{2}}{N\times max\left([PIF{]}_{m,p}^{*}\right)}+\sum_{i=1}^{N}\frac{{\left({\left[PhyA\right]}^{*}(i)-[T_{PhyA}]\;(i)\right)}^{2}}{N\times max({\left[PhyA\right]}^{*})}\\ & \;\;\;+\sum_{i=1}^{N}\frac{{\left({\left[PhyB\right]}^{*}(i)-[T_{PhyB}]\;(i)\right)}^{2}}{N\times max({\left[PhyB\right]}^{*})}+\sum_{i=1}^{N}\frac{{\left({\left[Cry1\right]}^{*}(i)-[T_{Cry1}]\;(i)\right)}^{2}}{N\times max({\left[Cry1\right]}^{*})}. \end{array} \end{array}$$

where the superscript * denotes the reference profiles, subscripts m and p represent the mRNA and protein, the notation ‘max’ indicates the maximum value of the reference profiles and N denotes the total simulation time points.

The references profiles of CL, P97, P51, EL and PIF genes profiles are taken from De Caluwé *et al.* (2016), and the reference values of phyA, phyB and cry1 are taken from Ohara *et al.* (2015a). The details about model MATLAB code are available in the Data Availability section.

### 3.2 PRC calculation

**Figure 2.**
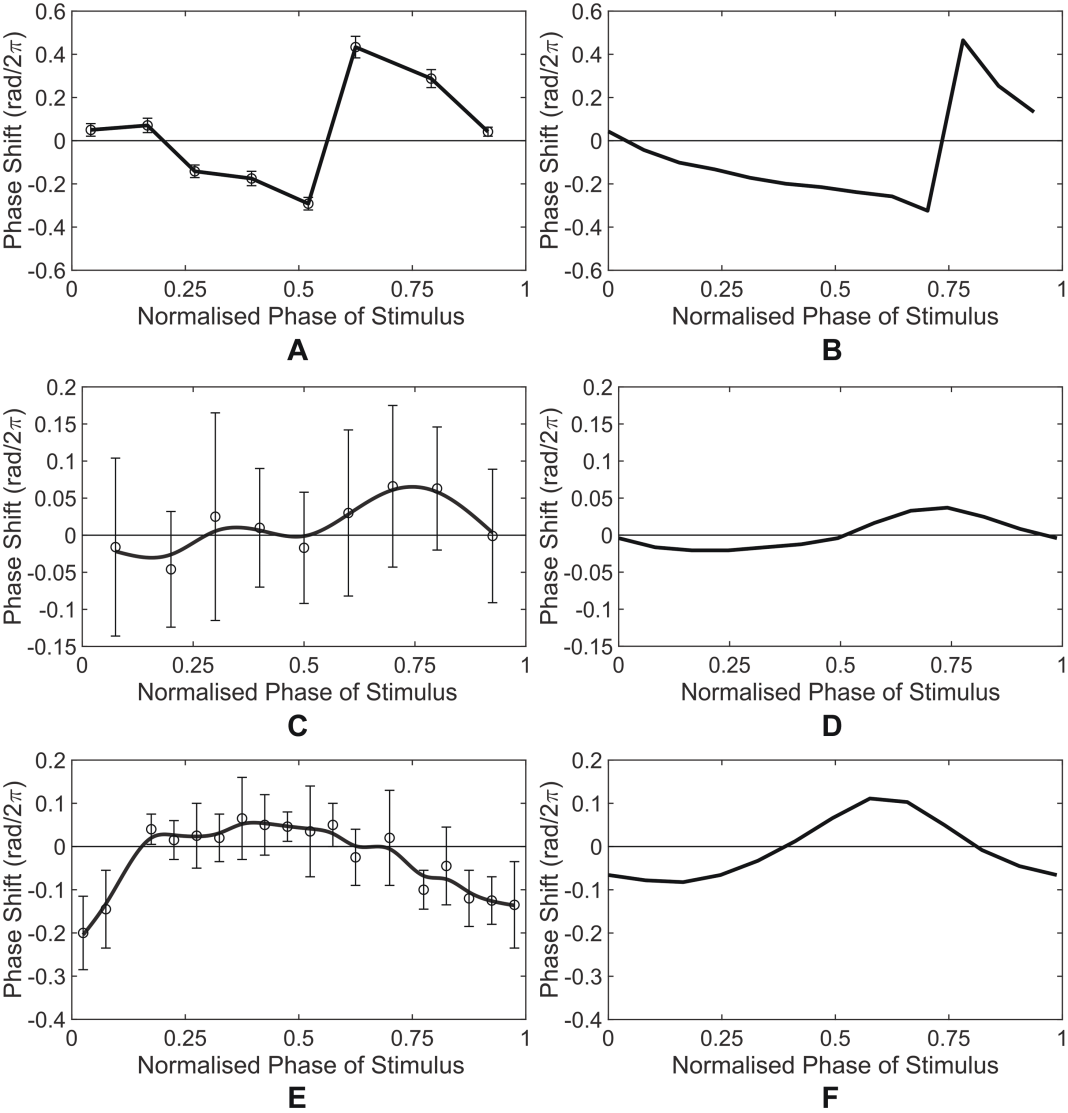
Comparison between experimental (left column) and simulated (right column) PRCs for Tests I–III. (A and B) Test I: Red Pulse; (C and D) Test II: Add Red; (E and F) Test III: Dark Pulse. The experimental PRCs were adapted from Ohara *et al.* (2015a).

The computation of the PRC (Figs 1 and 2) follows the approach presented in Ohara *et al.* (2015a), where light stimulus described in Table 2 is given to our model and, this simulation is repeated by varying the stimulus time over one circadian period (~24 h) to obtain the phase shift as a function of the phase of stimulus. The collection of this functional relationship forms the PRC.

**Table 2.** Light conditions for the PRC tests following Ohara *et al.* (2015a). The source of the experimental PRCs is given in the Reference column. Test I: Red Pulse—Turn on red light for 1 h under constant darkness. Test II: Add Red—Increase the red light intensity for 2 h under constant red light. Test III: Dark Pulse—Turn off the light for 2 h under constant red light. Test IV: Blue Pulse—Turn on the blue light for 1 h under constant darkness. Test V: Turn Blue—Switch the red light to blue light for 2 h under constant red light. Test VI: Add Blue—Turn on blue light for 2 h under constant red light while maintaining the red light on during light stimulus. These six tests are, respectively, termed ‘red-pulse’, ‘add-red’, ‘dark-pulse’, ‘blue-pulse’, ‘turn-blue’ and ‘add-blue’ as done in Ohara *et al.* (2015a).

Test no.	Condition	Background light		During stimulus			Reference
		Colour	Intensity (μmol·m^−2^s^−1^)	Colour	Intensity (μmol·m^−2^s^−1^)	Duration (h)	
I	Red Pulse	Dark	0	Red	40	1	Covington *et al.* (2001)
II	Add Red	Red	80	Red	160	2	Ohara *et al.* (2015b)
III	Dark Pulse	Red	80	Dark	0	2	Fukuda *et al.* (2008)
IV	Blue Pulse	Dark	0	Blue	25	1	Covington *et al.* (2001)
V	Turn Blue	Red	80	Blue	80	2	Ohara *et al.* (2015b)
VI	Add Blue	Red	80	Blue and Red	80	2	Ohara *et al.* (2015b)

Denoting ϕ as the normalized phase of stimulus, this can be calculated using the following equation.


$$\begin{array}{l} \phi =\frac{t_{\delta }-t_{r}}{T_{p}} \end{array}$$ (27)

where $t_{\delta }$ is the time of the light stimulus, $t_{r}$ is the peak time of the circadian gene expression of interest (in our case, CL mRNA) right before the light stimulus, $T_{p}$ is the free-running period and $t_{r}\leq t_{\delta }\leq t_{r}+T_{p}$. The free-running period is the duration of one complete circadian cycle under constant light conditions (i.e. either continuous light (LL) or continuous dark (DD)). The phase shift due to light stimulus is defined as follows:


$$\begin{array}{l} \Delta \phi =\frac{\Delta t_{d}}{T_{p}} \end{array}$$ (28)

where $\Delta t_{d}$ is the peak time difference between stimulated and non-stimulated circadian rhythm. To ensure minimal transient effect, light stimulus was given after 200 h of circadian rhythm and the phase shift was computed at the 18th circadian cycle after the light stimulus.

### 3.3 Calculation of the area of the ratio between phase-advance and phase-delay regions (*A/D* ratios)

Following the approach used in Ohara *et al.* (2015a), the *A/D* ratio is used to provide quantitative comparison of the simulated and the experimental PRCs. We first used zero-crossing to identify the phase-advance and phase-delay regions of the PRC curve. Then, the areas of these regions were calculated using MATLAB function trapz, which uses trapezoidal integration method. Finally, the *A/D* ratios can be computed using the following equation:


$$\begin{array}{ll} A/D=\; & Area\;of\;the\;phase\;(advance\;region)/\\ & Area\;of\;the\;phase\;(delay\;region) \end{array}$$

A symmetrical PRC has *A/D* = 1, while a asymmetrical PRC has *A/D* < 1 or *A/D* > 1. For asymmetrical PRC, *A/D* > 1 has larger phase-advance than phase-delay and vice versa for *A/D* < 1.

### 3.4 Experimental set-up for hypocotyl length measurement

Seedling of the wild-type *Arabidopsis* (Col-4) was grown hydroponically in a grow tent (Budda Room Grow Tent KitBox Silver Mylar Hydroponic Bud Dark Indoor) and the temperature of the grow tent was maintained between 18 and 22 °C. The plants were exposed to red, blue and mixed (red and blue) lights. The light source is provided by DZLight LED Light, Timer Function (Auto ON/OFF) 18-W Dual Head Horticultural Growing Lamps with 360 Degree Adjustable Gooseneck for Indoor Plants Greenhouse Hydroponics Gardening Office. For individual red and blue light, an intensity of 26.6 μmol·m^−2^s^−1^ is used, while for mixed light both red and blue light with intensity of 26.6 μmol·m^−2^s^−1^ are used for three different photoperiods; 2L22D, 4L20D and 6L18D for 10 days. The pictures of hypocotyl were taken on day 10 (Fig. 4B) and the hypocotyl lengths were measured via image processing using ImageJ software. Measurement of 10 hypocotyl lengths was taken to observe the variability. The average and standard deviation values were shown in (Fig. 4C and Table 3).

**Table 3.** Summary of hypocotyl length.

	Light quality	Hypocotyl length (mm)		
		6L18D	4L20D	2L22D
Simulated	Blue	8.21	11.98	15.88
	Red	8.86	12.67	16.14
	Mixed	8.50	12.27	16.01
Measured	Blue	5.13	6.14	8.77
	Red	7.01	9.42	10.60
	Mixed	5.59	6.95	9.20
Simulated (normalized)	Blue	5.13	6.14	8.77
	Red	7.01	9.42	10.60
	Mixed	5.97	7.52	9.69

**Figure 3.**
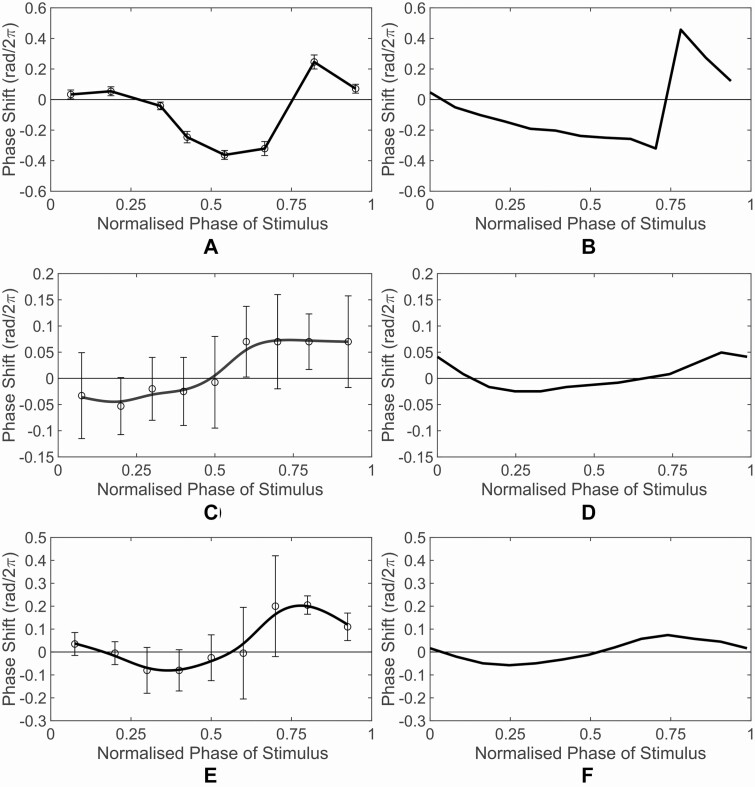
Comparison between experimental (left column) and simulated (right column) PRCs for Tests IV–VI. (A and B) Test IV: Blue Pulse; (C and D) Test V: Turn Blue; (E and F) Test VI: Add Blue. The experimental PRCs were adapted from Ohara *et al.* (2015a).

**Figure 4.**
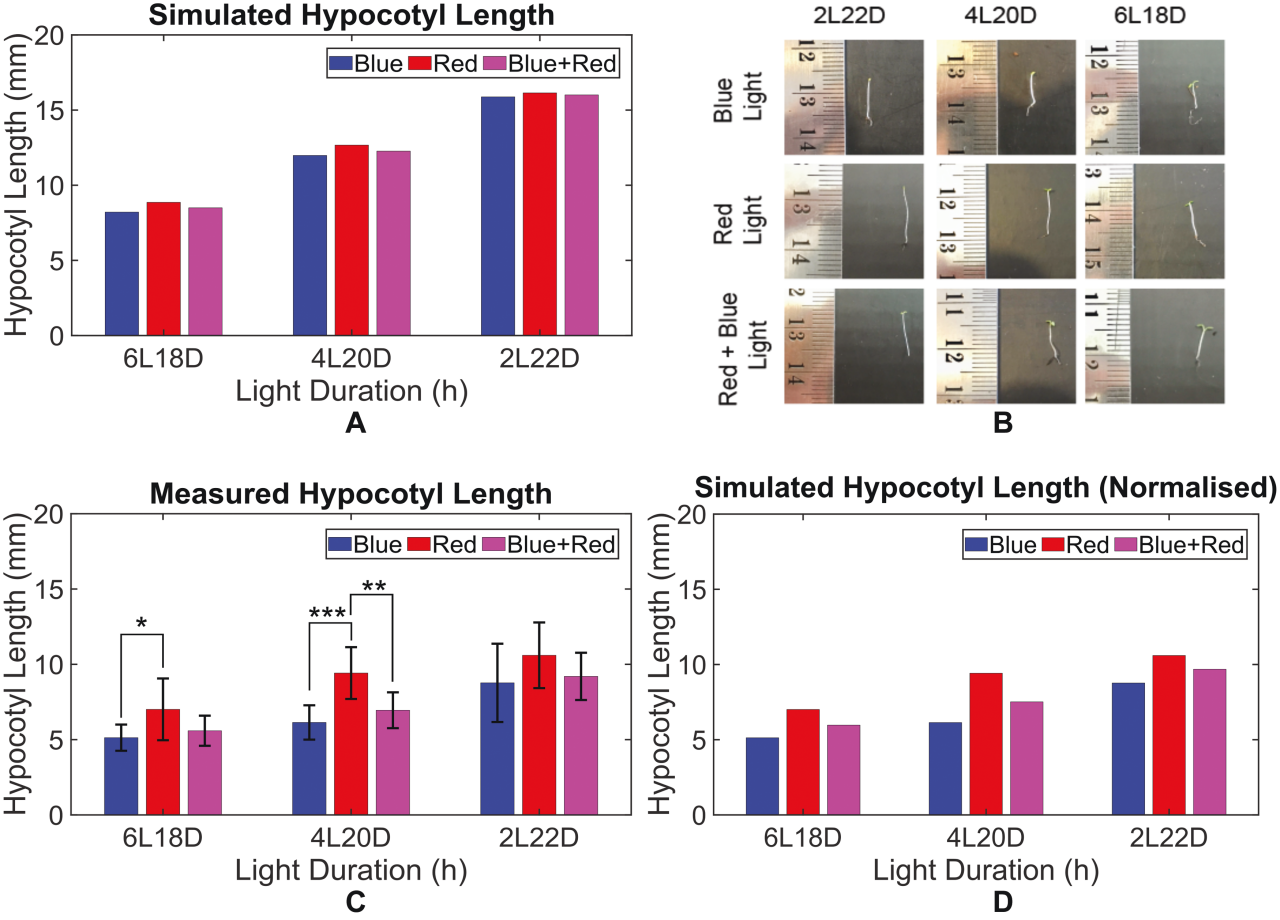
(A) Simulated hypocotyl length. (B) Hypocotyl of *Arabidopsis* WT Col-4 for different light quality conditions and photoperiods after 10 days. (C) Average measurement of 10 hypocotyl length with error bars denoting standard deviation and asterisk denoting statistical test. (D) Simulated hypocotyl length with min–max scaling technique.

### 3.5 Min–max normalization of hypocotyl length calculation

To facilitate a better quantitative comparison of the simulated and the experimentally measured hypocotyl length, min–max normalization technique is used in scaling the simulated data (Fig. 4A and Table 3). The simulated data are normalized (Fig. 4D and Table 3) within the range of experimental data for each photoperiods by using the following equation:


$$\begin{array}{l} x_{scaled}=\frac{\left(max_{exp}-min_{exp})(x-min_{sim}\right)}{\left(max_{sim}-min_{sim}\right)}+min_{exp} \end{array}$$

where x is the simulated data that need to be normalized, while $max_{exp}$ and $min_{exp}$ denote the maximum and minimum experimental values, respectively.

### 3.6 Statistics

In this study, asterisks indicate significant *P*-values as follows: *P < 0.05; **P < 0.01; ***P < 0.001. Data across multiple experiments are shown as mean ± SD. Student’s *t*-test was performed in Fig. 4C using MATHEMATICA 11.0.

## 4. RESULTS

### 4.1 Validation of model under free-running conditions and mutant genotypes

The first step in any PCS model validation is to make sure that the gene expressions oscillate in a self-sustaining manner under different free-running conditions (i.e. constant light (LL) and constant dark (DD) conditions) with a reasonable period. As shown in the ‘Wild type’ part of Table 1 and **Supporting Information—Fig. S1**, the simulated period under LL and DD conditions match the reported experimental period with good accuracy.

**Table 1.** Comparisons between experimental and simulated period under different mutant genotypes and light conditions. LL and DD represent constant light and dark, respectively. The LL condition is simulated in the model by turning on both red and blue lights with both their intensities set to 40 μmol·m^−2^s^−1^. $\delta P=P_{exp}-P_{sim}$ represents the difference in periods obtained via experiments ($P_{exp}$) and simulations ($P_{sim}$). ‘arr’ and ‘OX’ denote arrhythmicity and overexpression, respectively. The average simulated period is computed using the MATLAB function findpeaks and to minimize any transient effect, only the simulated data obtained after the first 200 h are used for the average period calculation.

Wild type		Experimental period (h)	Simulation period (h)	δP (h)	Reference
Constant light		24.6	24.6	0	Farré *et al.* (2005)
Constant dark		25.9	25.7	+0.2	Somers *et al.* (2000)
Mutant	Light condition	Experimental period (h)	Simulation period (h)	δP (h)	Reference
Δlhy/cca1	LL	19.7	25.7	−6.0	Lu *et al.* (2009)
Δtoc1	LL	21.0	24.0	−3.0	Strayer *et al.* (2000)
Δprr7	LL	25.9	25.8	+0.1	Farré *et al.* (2005)
Δelf3	LL	arr	arr	—	McWatters *et al.* (2000)
PRR5-OX	LL	22.7	24.8	−2.1	Baudry *et al.* (2010)
ELF3-OX	LL	26.8	25.1	+1.7	Herrero *et al.* (2012)
Δprr7	DD	25.8	25.7	+0.1	Farré *et al.* (2005)
Δelf3	DD	25.4	25.7	−0.3	Covington *et al.* (2001)
ELF3-OX	DD	29.5	25.7	+3.8	Herrero *et al.* (2012)

We next compare the simulated periods of the PCS model under different mutant genotypes against their respective periods reported in the literature, which is an important criteria for the model validity (see, e.g. Zeilinger *et al.* 2006; Relógio *et al.* 2011; Kim and Forger 2012). The simulation of the knockdown and overexpression mutants are done by considering a 50% reduction of the transcription rate of the targeted gene profile following De Caluwé *et al.* (2016) and adding a constant to the overexpressed genes following Foo *et al.* (2016), respectively. If we consider the predicted periods of these mutant genotypes with |*δP*| ≤ 4 h to be acceptable following the justification used in Foo *et al.* 2016, 2020, then the results shown in the ‘Mutant’ part of Table 1 and **Supporting Information—Fig. S1** demonstrate that the accuracy of our model is remarkable; predictions are correct except for only one scenario, i.e. ∆*lhy/cca1*, which deviates from |*δP*| by 2 h.

### 4.2 Validation of model using PRC

To further ensure that our proposed modification is rightly done, we evaluate our model against the experimental PRC following the same validation tests carried out in Ohara *et al.* (2015a) (Table 2; see also Section 3.2).

As shown in Figs 2 and 3, in general, the simulated PRCs obtained from our model in all six tests are in good agreement with the experimental PRCs in terms of the magnitude and shape. Only for Test III: Dark Pulse, there is a small inconsistency: the experimental PRC has a flatter peak ranging from the normalized phase of stimulus of 0.25 to 0.6 while the simulated PRC peaks at around normalized phase of stimulus of 0.6.

To provide a quantitative comparison of the PRCs obtained from our model with the experimental one, following the approach used in Ohara *et al.* (2015a), a comparative analysis using the area of the ratio between the phase-advance and phase-delay regions (*A/D*) of the PRCs are calculated. The *A/D* ratio provides information regarding the symmetrical property of the PRC with *A/D* = 1 indicating a symmetrical PRC, while *A/D* < 1 or *A/D* > 1 indicating a asymmetrical PRC (see Section 3.2 for more details). The results of the *A/D* ratios are shown in **Supporting Information—Table S2**. The value of the *A/D* ratios from our model is similar to the value of *A/D* ratios of the experimental PRCs for majority of the tests, while the direction (i.e. *A/D* < 1 or *A/D* > 1) of the *A/D* ratios from our model is in agreement with the experimental one except for Test I: Red Pulse condition. This is encouraging given that the reported *A/D* ratios calculated using the PCS model of Ohara *et al.* (2015a) are close to unity for all conditions (see also **Supporting Information—Table S2**). Taken together, these results further validate the accuracy of our model with improvement in the PRC generation as compared to Ohara *et al.* (2015a).

### 4.3 Hypocotyl growth under different light quality conditions

With the model accurately capturing the effect of light qualities on PCS (Figs 1 and 2), we next explore the effect of light qualities on plant growth and development. Specifically, we simulated the model under blue, red and mixed (blue and red) light conditions across three different photoperiods, i.e. 2 h light, 22 h dark (2L22D), 4 h light, 20 h dark (4L20D) and 6 h light, 18 h dark (6L18D) for 10 days. These three photoperiods were considered because hypocotyls tend to be longer with shorter photoperiod and the lengths do not vary much for light duration greater than 8 h (Seaton *et al.* 2015). Note that the intensity for the individual red and blue light condition is set to 26.62 μmol·m^−2^s^−1^. For mixed light condition, we have set both red and blue light intensities to 26.62 μmol·m^−2^s^−1^, resulting in the total intensities of 53.24 μmol·m^−2^s^−1^.

The simulated hypocotyl lengths are shown in Fig. 4A and Table 3. Across all three light quality conditions, hypocotyls elongate more as the light duration decreases. This is consistent with experiments where shade-intolerant plants have adapted to low light conditions through increased growth when low light is detected such as the case of 2L22D (Botterweg-Paredes *et al.* 2020). Furthermore, the trend of the hypocotyl length associated with individual blue and red lights is in agreement with experimental findings (Hu *et al.* 2013). Interestingly, when comparing the hypocotyl lengths subject to mixed red and blue light qualities, we observe that across the three photoperiods, the hypocotyl length is longer in the following light quality order; blue, mixed and red. Notably, the simulated hypocotyl length under mixed light qualities is shorter than red light but longer than blue light, consistent with the notion of a competitive binding of the different photoreceptors to COP1 (Fig. 1B) and their likely differences in effectiveness of disrupting COP1 activity **[see****Supporting Information—Table S1****]**.

To confirm the dependency of hypocotyl length on light qualities and duration simulated by our model (Fig. 4A), we performed an experiment to measure the hypocotyl lengths under three different light quality conditions across different photoperiods as shown in Fig. 4B (see also Section 3). The experimentally measured hypocotyl lengths are shown in Fig. 4C and Table 3. The results in Fig. 4C show that the hypocotyl lengths across different light quality conditions are increasing in the order of blue, mixed and red light for all three photoperiods, in which the trend is consistent with our simulation result (Fig. 4A). Although the proposed model correlates well with the experimental data albeit in a qualitative manner, there are quantitative differences: overall the model predicts longer hypocotyl length with smaller differences across different light quality conditions compared to the experiments. This quantitative mismatch would indicate that there might be hidden interactions related to the hypocotyl growth that are not incorporated into the current model and this issue will be addressed as part of our future works (see Section 5).

To illustrate how our model can accurately predict the hypocotyl length if this quantitative mismatch can be circumvented, we normalized our simulated hypocotyl using min–max scaling technique (Fig. 4D and Table 3, see also Section 3.5 for details), which has been commonly used to ensure that the normalized data range of different scale (see, e.g. Mateu *et al.* 2016). Indeed, after normalization, the simulated hypocotyl lengths are consistent with the measured hypocotyl lengths quantitatively as well as qualitatively across all light quality and duration conditions.

## 5. DISCUSSIONS AND CONCLUSIONS

In this study, we have presented a mathematical model of PCS to characterize the effect of light qualities on plant growth viz hypocotyl elongation. In order to effectively account for the effect of red and blue lights on hypocotyl growth, we have modified an existing PCS model by adding three photoreceptors. The developed model also takes into account recent results on the role CL proteins play in repressing certain genes (Fogelmark and Troein 2014; Adams *et al.* 2015). More importantly, the interactions of the photoreceptors with a key photomorphogenic regulator, COP1, were also incorporated into the model, which supports the notion of competitive interaction between phy and cry photoreceptors.

Our model is capable of reproducing periods under free-running and different mutant genotypes (Table 1) as well as experimental PRCs under different light quality stimulus conditions (Figs 2 and 3 and **Supporting Information—Table S2**). In particular, the model predicted that red and blue light receptors, phys and cry1, competitively bind with COP1 under mixed light condition (i.e. red and blue), resulting in the more hypocotyl elongation under red light condition than under mixed light condition. This prediction was confirmed by our experiment (Fig. 4).

While the difference of the hypocotyl growth across different light qualities may not seem significant, our model does capture the relevant biological interpretation of the proposed competitive binding mechanism. In **Supporting Information—Fig. S2**, we show that without the competitive binding mechanism, the simulated hypocotyl growth from our model display no difference in hypocotyl growth across different light qualities (i.e. the hypocotyl growth is independent of light qualities). While our model cannot capture the experimental data quantitatively, it captures the data qualitatively unlike the model without competitive binding. This indicates that competitive binding is critical for capturing the observed experimental behaviour under different light qualities. Also, it is worth mentioning that these hypocotyl growths from our experiment were not used in obtaining our model parameters and the simulated hypocotyl growth is a direct result of incorporating the competitive binding mechanism.

The quantitative mismatch of hypocotyl length between simulation and experiment (Fig. 4A and C) could be attributed to the absence of experimental data in obtaining the model parameters or it indicates that there could be hidden mechanisms that were not included in the current light module. Some potential candidates would be gating for light, which reduces photosensitivity of the circadian clock depending on the circadian phase (Miyake *et al.* 2000; Geier *et al.* 2005; Pulivarthy *et al.* 2007; Kiessling *et al.* 2014; Kim *et al.* 2019), and adaptation for light, which reduces photosensitivity depending on light duration (Ding *et al.* 1997; Jagannath *et al.* 2013; Cao *et al.* 2015). To address our model limitation, incorporation of gating and adaptation functions into the current light module as done in (Bellman *et al.*, 2018; Kim et al., 2019) (e.g. using multiplicative light module) would be an interesting future work.

As the developed model is fairly accurate, our results open up the possibility of developing similar model for other physiological outputs such as flowering (Foo and Kim 2014; Johansson and Staiger 2015), photosynthesis (Dodd *et al.* 2005) and root growth (Seaton and Krishnan 2016; Fung-Uceda *et al.* 2018; Lochocki and McGrath 2021). The development of such models would be of great interest particularly from speed breeding point of view as more phenotypic characteristics related to plant growth can be predicted in advance.

The protocol of speed breeding is currently performed based on the expert knowledge-based approach. This could involve investigation of numerous combinations of light qualities and photoperiods to identify the optimal protocol, which is time- and resource-consuming. To circumvent this, the *in silico* approach using highly predictive model could play the critical role. Specifically, the model prediction could assist experts in focusing on certain promising set of light qualities and photoperiods combinations, ultimately leading to drastically cutting the experimental time and resources (see, e.g. Pereira *et al.* 2021). In this regard, our model can be considered as an important step towards model-based plant productivity enhancement research. This will contribute immensely to increase the food production to feed the ever-increasing world population.

## SUPPORTING INFORMATION

The following additional information is available in the online version of this article—

**Figure S1.** Comparisons between experimental and simulated period under different mutant genotypes and light conditions based on Table 1.

**Figure S2.** Simulated hypocotyl length without competitive binding.

**Table S1.** Estimated model parameters.

**Table S2.** Comparison of area of the ratio between the phase-advance and phase-delay regions (*A/D*) between experimental, simulation and Ohara *et al.* (2015a) model.

diac001_suppl_Supplementary_Materials

## Data Availability

The MATLAB codes of model for generating the simulation are publicly available at https://github.com/mathiasfoo/hypocotylmodel.
