# Supplementary figures and images for: Modelling of plant circadian clock for characterizing hypocotyl growth under different light quality conditions
Source: In Silico Plants. 2022 Feb 2;4(1):diac001. doi: 10.1093/insilicoplants/diac001 (PMC8963510; doi:10.1093/insilicoplants/diac001)

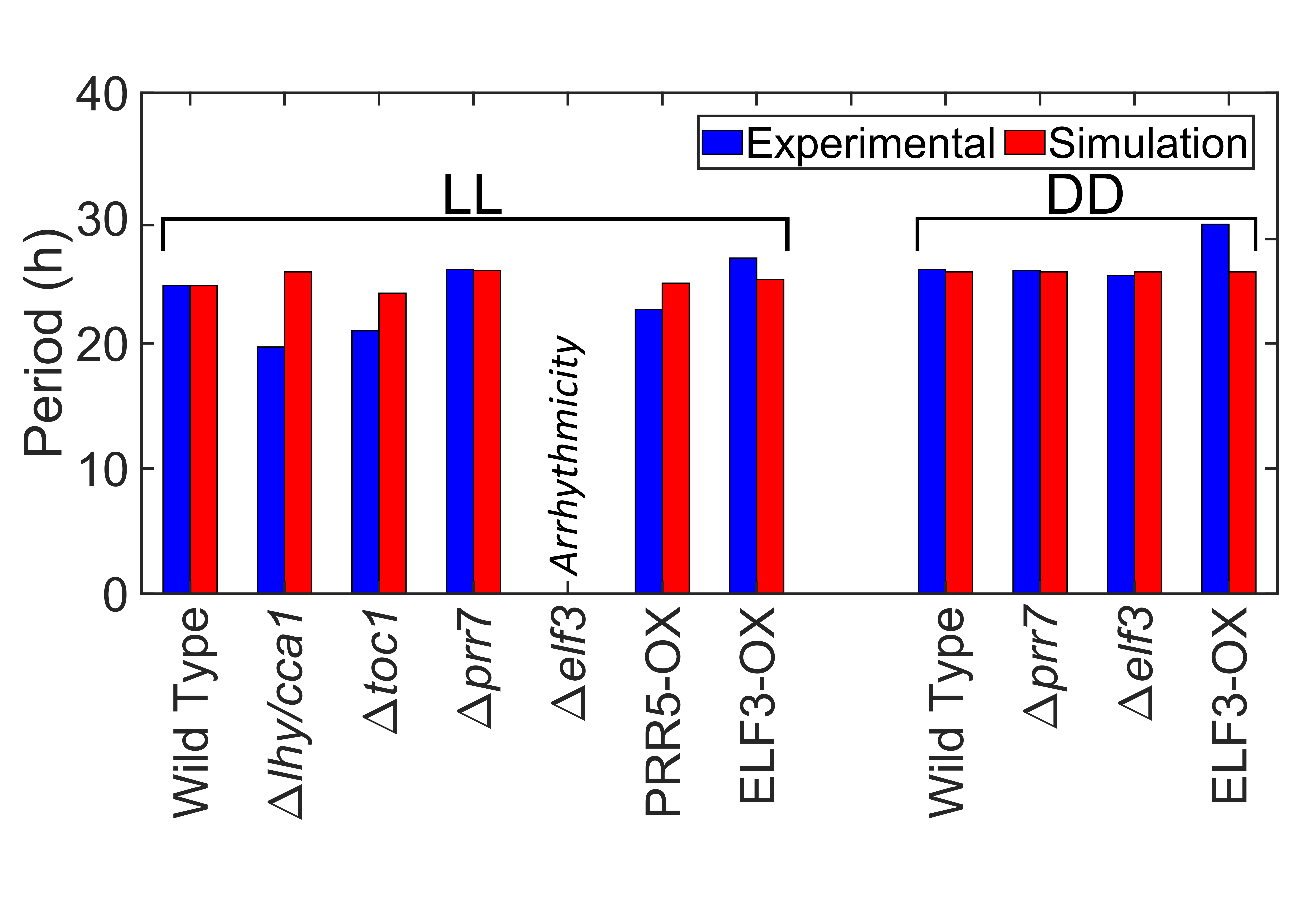

Supplement: diac001_suppl_Supplementary_Materials [file diac001_suppl_supplementary_materials.zip › diac001_suppl_Supplementary_Figure_S1.png]

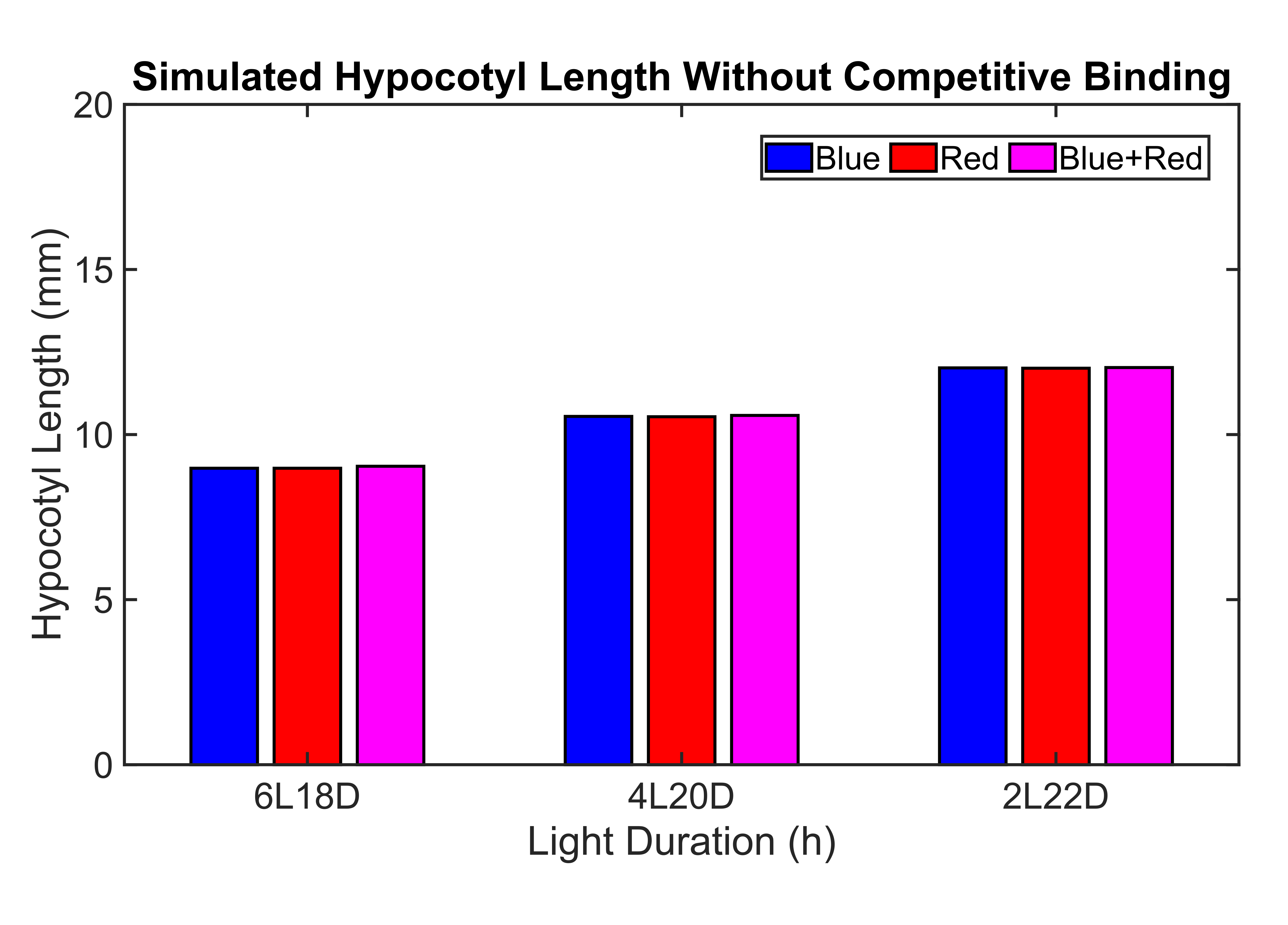

Supplement: diac001_suppl_Supplementary_Materials [file diac001_suppl_supplementary_materials.zip › diac001_suppl_Supplementary_Figure_S2.png]
